# Enhancing Aging and Ending Ageism: *JAMA Network Open* Call for Papers

**DOI:** 10.1001/jamanetworkopen.2021.17621

**Published:** 2021-06-01

**Authors:** Sharon K. Inouye, Ishani Ganguli, Elizabeth A. Jacobs

**Affiliations:** Department of Medicine, Beth Israel Deaconess Medical Center, Harvard Medical School, Boston, Massachusetts; Marcus Institute for Aging Research, Hebrew SeniorLife, Boston, Massachusetts; *JAMA Network Open*, Chicago, Illinois; Department of Medicine, Brigham and Women’s Hospital, Harvard Medical School, Boston, Massachusetts; *JAMA Network Open*, Chicago, Illinois; MaineHealth and Maine Medical Center Research Institute, Scarborough; Departments of Medicine and Population Health, The University of Texas, Austin; *JAMA Network Open*, Chicago, Illinois

The US National Academy of Medicine^1^ and the World Health Organization^2^ have identified aging and ending ageism as urgent issues for our era. In the past century, human life expectancy increased nearly 2-fold, a profound increase exceeding the number of years added in all prior millennia combined. Globally, more than 600 million people are aged 65 years or older, and this number is expected to exceed 1.6 billion by 2050, representing nearly 20% of the world’s population. The population of individuals aged 80 years or older will increase more than 3-fold, reaching nearly half a billion globally.^3^ This gain in life expectancy has been driven by advances in public health, sanitation, socioeconomic development, public education, and health care. It is an unparalleled human achievement that presents both extraordinary challenges and opportunities.

Gains in life expectancy coupled with declines in fertility have substantially shifted population demographics, particularly in industrialized countries, where the proportion of older individuals may outnumber the young, producing critical economic, social, and health care challenges. Societies are increasingly forced to grapple with the rising health care expenditures and long-term care needs of multimorbidity and disability that are associated with aging. Age is the strongest risk factor for many chronic diseases, including heart disease, stroke, cancer, and dementia^4^—all of which may require extensive health care and long-term care services.

The COVID-19 pandemic placed additional stress on societal and health care delivery systems worldwide, exposing deeply entrenched structural ageism and leading to high rates of morbidity and mortality among older populations.^5^ Age-associated inequities in health care access, delivery, and outcomes revealed how unprepared health care systems are to meet the needs of a rapidly aging society. These circumstances have prompted the United Nations, World Health Organization, World Economic Forum, and Global Age-Watch to call for prioritizing medical, scientific, social, and financial preparedness for population aging as a global imperative.^6^

Most aging research to date has focused on risk factors, diseases, impairments, and health care outcomes that increase with age, as well as the challenges that arise with aging. Moreover, medical research has often reflected the disease- and cure-focused nature of our health systems, rather than the whole-human, person-centered approach that is essential for optimal care of older adults. Importantly, the current emphasis distorts the reality of aging, largely ignoring many of the potential benefits for individuals and communities. The present generation is aging with better health and functioning than any generation before. Most older adults retain the ability to work well into their 80s.^7,8^ While some cognitive functions decline with age, some functions, such as complex decision-making (which requires expertise and experience), do not peak until age 70 years or older.^9,10^ Some studies, such as a 2010 study by Charles and Carstensen,^11^ have found that older persons report significantly less stress and worry and significantly more happiness and life satisfaction than younger adults. Acceptance, adaptability, and resilience are common traits seen with aging. Leading economists have identified the growing older population as an untapped resource of wealth and potential societal contribution, providing a “new demographic dividend.”^12^

*JAMA Network Open* is issuing a call for papers to contribute to the evidence base to build the health and social care system needed to provide better health and health care for older adults and to eliminate ageism. We seek rigorous, high-quality scientific evidence to address critical gaps in knowledge that will advance our understanding in priority areas. These priority areas include^1^: ageism and building an antiageist, equitable health care infrastructure that addresses disparities and social determinants of health^2^; geriatric workforce development, education, training, and support^3^; prevention of geriatric syndromes (eg, delirium, falls, frailty, incontinence), and chronic conditions and diseases related to aging^4^; broad public health interventions for healthy aging^5^; new geriatric models of care that provide goal-directed, person-centered care, maximize function, and address advance directives^6^; long-term care with integration of social care and health care systems, integration of families and informal caregivers, and enhancement of aging in place^7^; policy and financing reforms to improve care access and quality across settings for older adults; and national and international comparisons of health care approaches for older adults.^8^

Our Instructions for Authors^13^ list the various study designs and reporting requirements for articles suitable for *JAMA Network Open*. Randomized and other controlled intervention trials are needed to increase knowledge in this area. Rigorous observational studies using cohort, case-control, and pragmatic designs are important when interventional studies are not feasible. Synthesis studies, including systematic reviews and meta-analyses, are useful for bringing clarity to existing literature, and evidence-based consensus statements and clinical practice guidelines that will help improve clinical practice are welcome. Note that *JAMA Network Open* does not publish narrative review articles, case reports, or unsolicited opinion articles.

All favorable research manuscripts undergo peer review, including statistical review. All articles accepted for publication will be eligible to have accompanying Invited Commentaries published by experts in the field and will be published quickly. In addition, all articles will be featured on the *JAMA Network Open* and JAMA Network journals’ websites. All *JAMA Network Open* articles are indexed in MEDLINE, Science Citation Index Expanded, Scopus, and other databases. The journal has broad reach through press releases and coverage by major news media and social media. Please see the journal’s Instructions for Authors for additional information on manuscript preparation and submission.^13^ This call for papers is open now, and we encourage submissions through January 31, 2022, and beyond.

Through this call, we hope to contribute to the evidence base to eliminate ageism; redesign and build an infrastructure for better health with aging; maximize health, productivity, and quality of life throughout late life; and achieve the longevity dividend: “the gift of long life.”^14^ As stated by the US National Academy of Medicine in their Vital Directions for Health initiative: “If these priorities are addressed proactively, an infrastructure can be created that promotes better health and equitable, goal-directed care that recognizes the preferences and needs of older adults.”^1^ If we can find ways to value, engage, and take good care of our elders—who include many of the most at-risk, sick, and disabled in society—we will create a world that is equitable for all.
